# The visual cortex in the blind but not the auditory cortex in the deaf becomes multiple-demand regions

**DOI:** 10.1093/brain/awae187

**Published:** 2024-06-12

**Authors:** Hasan Duymuş, Mohini Verma, Yasemin Güçlütürk, Mesut Öztürk, Ayşe B Varol, Şehmus Kurt, Tamer Gezici, Berhan F Akgür, İrem Giray, Elif E Öksüz, Ausaf A Farooqui

**Affiliations:** Department of Psychology, Bilkent University, Ankara, 06800, Türkiye; Department of Psychology, Ankara Yildirim Beyazıt University, Ankara, 06760, Türkiye; Department of Neuroscience, Bilkent University, Ankara, 06800, Türkiye; Aysel Sabuncu Brain Research Center, Bilkent University, Ankara, 06800, Türkiye; Sign Language Program, TÖMER, Ankara University, Ankara, 06100, Türkiye; Sign Language Program, TÖMER, Ankara University, Ankara, 06100, Türkiye; Department of Neuroscience, Bilkent University, Ankara, 06800, Türkiye; Department of Psychology, Ankara Yildirim Beyazıt University, Ankara, 06760, Türkiye; Department of Neuroscience, Bilkent University, Ankara, 06800, Türkiye; Department of Neuroscience, Bilkent University, Ankara, 06800, Türkiye; Department of Neuroscience, Bilkent University, Ankara, 06800, Türkiye; Department of Psychology, Ankara Yildirim Beyazıt University, Ankara, 06760, Türkiye; Department of Psychology, Bilkent University, Ankara, 06800, Türkiye; Department of Neuroscience, Bilkent University, Ankara, 06800, Türkiye; Aysel Sabuncu Brain Research Center, Bilkent University, Ankara, 06800, Türkiye; National Magnetic Resonance Research Center, Bilkent University, Ankara, 06800, Türkiye

**Keywords:** neuroplasticity, blind, multiple-demands network, cognitive control, deaf

## Abstract

The fate of deprived sensory cortices (visual regions in the blind and auditory regions in the deaf) exemplifies the extent to which experience can change brain regions. These regions are frequently seen to activate during tasks involving other sensory modalities, leading many authors to infer that these regions have started to process sensory information of other modalities. However, such observations can also imply that these regions are now activating in response to any task event, regardless of the sensory modality. Activating in response to task events, irrespective of the sensory modality involved, is a feature of the multiple-demands (MD) network. This is a set of regions within the frontal and parietal cortices that activate in response to any kind of control demand. Thus, demands as diverse as attention, perceptual difficulty, rule-switching, updating working memory, inhibiting responses, decision-making and difficult arithmetic all activate the same set of regions that are thought to instantiate domain-general cognitive control and underpin fluid intelligence.

We investigated whether deprived sensory cortices, or foci within them, become part of the MD network. We tested whether the same foci within the visual regions of the blind and auditory regions of the deaf activated in response to different control demands.

We found that control demands related to updating auditory working memory, difficult tactile decisions, time-duration judgments and sensorimotor speed all activated the entire bilateral occipital regions in the blind but not in the sighted. These occipital regions in the blind were the only regions outside the canonical frontoparietal MD regions to show such activation in response to multiple control demands. Furthermore, compared with the sighted, these occipital regions in the blind had higher functional connectivity with frontoparietal MD regions. Early deaf, in contrast, did not activate their auditory regions in response to different control demands, showing that auditory regions do not become MD regions in the deaf.

We suggest that visual regions in the blind do not take a new sensory role but become part of the MD network, and this is not a response of all deprived sensory cortices but a feature unique to the visual regions.

## Teaser

The fate of sensory brain regions when deprived of their usual sensory inputs can tell us about the extent to which experience can alter brain regions. Duymuş *et al.* show that visual regions in the blind, but not auditory regions in the deaf, become cognitive control regions and activate in response to any kind of control demand.

## Introduction

What becomes of sensory cortices when deprived of their sensory input is unclear. Visual regions in blind people have been seen to activate during diverse auditory, tactile, olfactory and language tasks, and auditory regions in deaf people have been seen to activate during visual, tactile, olfactory and language tasks.^1-9^ Existing accounts have typically interpreted these findings in sensory terms.^3,7,10-19^ Some have argued that these regions become crossmodal and develop different sensory specializations. Others have claimed that such activations are limited to the higher sensory regions and that these regions have not truly changed. These are either doing the same metamodal processing on a different sensory input, e.g. visual motion regions process auditory motion in blind people, or these regions were multisensory, hence supramodal, even in their non-deprived state, e.g. visual motion regions even in the sighted process auditory motion.^2,3,5,14,20-28^ This issue is thus of intense interest because it remains unclear whether deprived sensory regions have truly reorganized into performing different functions^29^ or are simply manifesting less prominent functions that were already inherent in them.^30,31^

Despite the tendency in existing studies to interpret the activation of deprived sensory cortices in sensory terms, it is unclear whether these activations are about sensory information *per se*. Activation of these regions, across all the very many studies done so far, always occurs in the context of some task; passive sensory stimulation in any modality does not activate these regions.^9,10^ The plethora of existing findings about the activation of deprived sensory cortices might not be instances of crossmodal activation, instead, these activations may be in response to all kinds of task-relevant events irrespective of their sensory modality. Given that activation in response to any task-relevant event, regardless of sensory modality, is a feature of multiple-demand (MD) regions,^32,33^ we investigated whether deprived sensory cortices, or foci within them, become part of the MD network.

MD regions are a set of frontoparietal regions that activate in response to any task-relevant event and to all kinds of control demands, irrespective of the sensory modality involved,^32,34-39^ and have been referred to variously as the cognitive-control, attentional, neuronal workspace and task-positive network.^40-43^ These regions instantiate domain-general cognitive control during any demanding task.^44-46^ The cardinal sign of these regions, because of which they were first identified,^47^ is the activation of a common set of loci in response to any control demand. Thus, demands as diverse as attention, working memory, response-conflict, inhibitory control, task-switching, control of memory, perceptual difficulty, action selection and decision-making activate the same loci in frontal and parietal cortices.^48-53^ Another feature of MD regions is that any task-relevant information (task-related stimuli, the focus of attention, contents of working memory, actions, goals, etc.) can be decoded from the activity patterns across the voxels of these regions.^54^ MD regions are functionally more connected with each other and are distinct from other brain networks, such as the default mode, language, motor and sensory networks of various modalities.^40^

Existing evidence already suggests that at least some foci within the occipital cortices in the blind might have become MD regions. Across different studies, any task-relevant information seems decodable from the occipital regions of the blind.^27,55^ Occipital cortices in the blind have increased functional connectivity with frontoparietal MD regions and not with other sensory regions, as observed in the sighted.^56-59^ Several studies have shown that foci within the primary visual cortices of the blind activate in response to higher cognitive demands of working memory, response inhibition, episodic memory recall and mathematical operations.^2,4,12,60-65^ However, it remains unclear whether these different demands activate the same foci, the key characteristic of MD regions.

**Figure 1:**
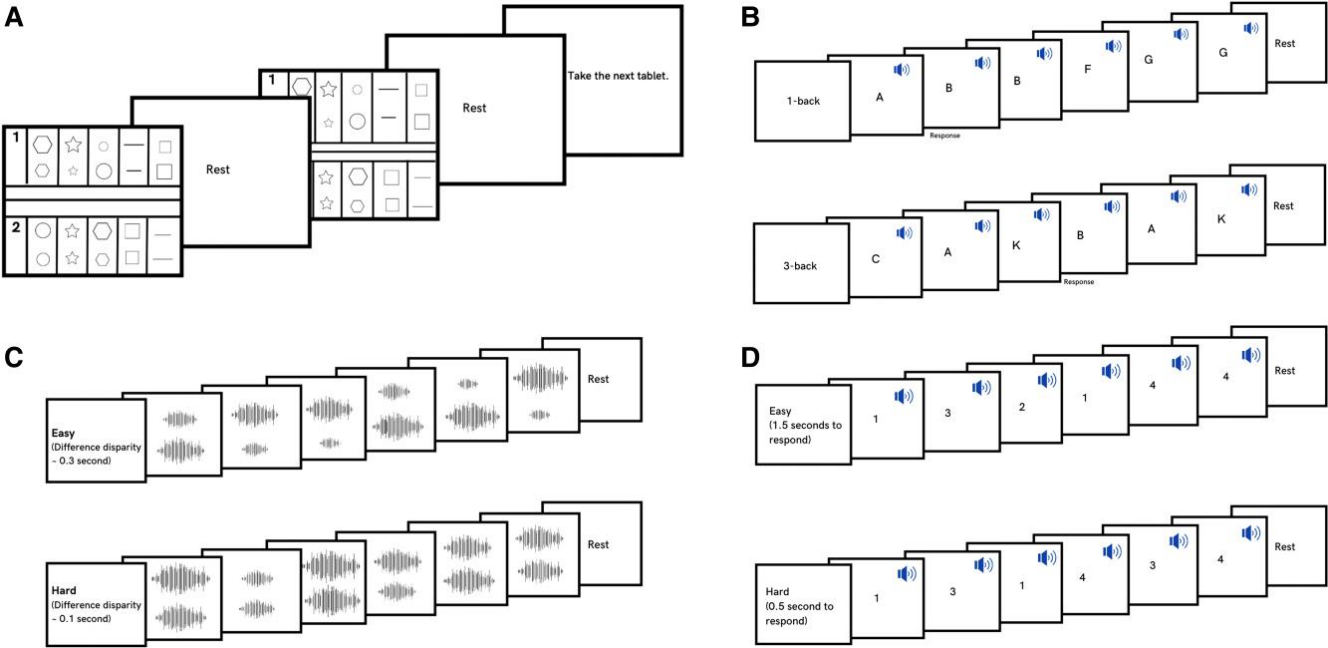
**Overview of the four functional MRI tasks: tactile decision-making, auditory working memory-updating, time-duration judgement and motor speed tasks.** All four tasks had an easy block and a hard block, each lasting 15–30 s. (**A**) Tactile decision-making: participants decided which of the two shapes was larger in size. The task was done using a plexiglass tablet that had an easy part and a hard part, each of which had five trials. Each trial involved a pair of shapes drawn with raised margins. Margins of shapes in easy trials were more raised, making them easier to perceive by touch. Furthermore, the difference in size between shapes of a pair was larger in easy trials. (**B**) Auditory working memory-updating: each block had 10 trials. Participants decided whether the letter they heard in a given trial was the same as one trial (on easy blocks) or three (on hard blocks) trials prior. (**C**) Time-duration judgement: each block had 10 trials. In each trial, participants heard two sequential tones of variable durations separated by a silent pause. They had to decide which of the two was longer in duration. Tones in easy blocks had larger differences in their durations, making it easier to discern the longer tone. (**D**) Motor speed: participants heard one of four numbers (1–4) and had to press the corresponding button. In easy blocks, they had 1.5 s to respond, whereas in hard blocks they had only 0.5 s. Deaf participants did a visual instead of auditory working memory-updating task (see the ‘Materials and methods’ section).

We investigated whether deprived sensory cortices or foci within them show the cardinal sign of MD regions, i.e. activation of the same foci in response to diverse kinds of control demands.^37,47^ We had blind, deaf and blindfolded sighted participants execute up to four different functional MRI (fMRI) tasks involving different modalities (Fig. 1). In the difficult versions of these four tasks, they had to: (i) update more working memory (WM) items; (ii) make difficult tactile decisions; (iii) execute speeded sensorimotor responses; and (iv) make more demanding time-duration judgments, respectively. We also carried out task-based functional connectivity analysis for the obtained data. We found that along with frontoparietal MD regions, the entire occipital cortices in the blind, but not in the sighted, activated in response to these diverse control demands and were functionally connected with MD regions. Auditory regions in the deaf, however, did not show such signs of MD regions.

## Materials and methods

### Participants

We tested 22 blind participants [nine females, mean age 29.8 years, standard deviation (SD) = 6.75]. Of these, 16 were congenitally blind, and six had acquired blindness between the ages of 6 and 18 years. Eighteen of them were right-handed and five left-handed. Medical records of all blind subjects reported no sight apart from faint light perception, and none had any neurological illness. Blind subjects were recruited through non-governmental blind foundations and personal contacts of two of the authors. The control group consisted of 20 participants (12 females, mean age 26.75 years, SD = 6.23). These were both sighted and hearing and had no history of neurological illnesses. Five of them were left-handed and the rest right-handed. A group of 10 early deaf participants (six females, mean age 35.4 years, SD = 4.41) also took part in the study. They were recorded either as being congenitally deaf or as having lost hearing before their first birthday. None of them had any history of neurological illnesses. All participants gave written consent before their participation. The study was approved by the Bilkent University Research Ethics Committee.

Sixteen of the blind participants completed all four tasks. Three completed three tasks (time-duration judgment or sensory motor speed in addition to auditory WM-updating and tactile decision-making), and three did only two tasks (auditory WM-updating and tactile decision-making). Control and deaf participants did only the WM-updating and the tactile decision-making tasks. Control participants executed their tasks blindfolded.

### Experimental design

Each of the four tasks involved alternating easy and hard blocks (Fig. 1). Blocks lasted 15–30 s and were separated by jittered rest periods (5–15 s). At the end of these rest periods, participants heard a cue that informed them about the nature (easy or hard) of the ensuing block and asked them to press a button when they were ready to start the block. Instead of hearing a cue, deaf participants saw a sign-language clip conveying this information. These different tasks were done in separate fMRI runs, each lasting 12–18 min. Experimental tasks were presented using Psychtoolbox in MATLAB.

#### Tactile decision-making task

Trials were presented through a plexiglass tablet that was placed on the chest or abdomen of the participant (Fig. 1A). Each tablet was bisected horizontally into an easy section and a hard section through three raised lines running through the middle. This helped participants to discern the boundary between the two sections via touch. Each section was further divided into five cells by four vertical lines spaced 3 cm apart. These cells represented individual trials and contained a pair of vertically aligned shapes drawn by raised lines. A tablet thus had an easy block and a hard block, each consisting of five trials. Participants executed a total of 10 easy and 10 hard blocks through 10 tablets.

The pair of shapes presented as part of one trial were always identical but differed in size and were drawn 2 cm apart. Shapes varied across different trials. In each trial, participants had to decide which of the two was larger in size and convey their answer with a button press (index finger for top shape; middle finger for bottom shape). They were then cued to move on to the next trial. After a block of five trials, a jittered rest period of 5–15 s followed. Participants were then asked to locate the tactile reference point of the next block (see later) and press a button. Given that a plexiglass tablet had two blocks, it had to be changed after every two blocks. This was done after the jittered rest period at the end of every second block.

The decision difficulty was manipulated by the difference in size between the shapes of a pair and the extent to which their margins were raised. For easy trials, the difference in size between the shapes of a pair was between 0.8 and 1 cm^2^. For hard trials, this difference was between 0.3 and 0.5 cm^2^. The extent to which the margins of the shapes were raised was lower in hard blocks, hence it was more difficult to discern them. The terms ‘easy’ and ‘difficult’ were embossed on the upper left edge of their respective sections in Braille for blind participants (below the Braille markers, numerical indicators were also embossed for deaf and control participants). These not only informed the participants about the nature of the ensuing block, but also served as a tactile reference point and oriented the participants about where to start their block of trials.

For the blind (and their sighted controls), prerecorded auditory instructions were used to convey instructions to the participants (to begin a block, change the plexiglass tablet, etc.; e.g. ‘Please spot the beginning of the easy block by placing your finger on the corresponding braille marker, then press a button when you are ready to proceed with trial 1’). For the deaf, prerecorded sign-language video clips were used for instructions. An experimenter present in the MRI room handed the plexiglass tablets to the participants and positioned them on their chest or abdomen. The order of blocks was randomized.

#### Working memory-updating task

This was an auditory n-back task (Fig. 1B). Trials were presented in blocks of 10. For easy and difficult blocks, participants did 1-back and 3-back tasks, respectively. In each trial, a letter was presented. For the 1-back task blocks, participants were to keep the first trial letter in mind and proceed to the next trial by pressing a button. From the second trial onwards, they were to respond if the letter they heard was the same as the previous trial (index finger for yes; middle finger for no). For the 3-back task blocks, the trial content was the same, but participants now had to remember the letters of the first three trials, and from the fourth trial onwards, they had to decide whether the current letter was the same as the one they heard three trials earlier. Easy and hard blocks alternated with each other, and their order was counterbalanced across participants. Participants executed a total of 10 easy and 10 hard blocks.

For deaf participants, the task was, likewise, organized into easy and hard blocks. Instead of an auditory n-back, they did a visual n-back task that involved a picture presented in each trial. The hard blocks involved 2-back and easy blocks 1-back tasks. Everything else was identical to the auditory n-back task.

#### Time-duration judgment task

The task was designed with a total of 20 blocks, comprising 10 alternating easy and hard blocks. Each block contained 10 trials. In each trial, participants were presented with a sequence of two distinct pure tones separated by a silent pause (Fig. 1C). One of the two tones was always of a longer duration than the other. Participants had to detect the longer tone (index finger for first tone; middle finger for second tone). The difference in the durations of the two tones of a trial was reduced to increase the difficulty of the hard block trials. This difference varied between 200 and 350 ms in easy trials and between 60 and 150 ms in hard trials. The intertrial interval was 1 s.

#### Sensorimotor speed task

The task was organized into alternating sequences of easy and hard blocks. Each block contained 8–12 trials. In each trial, participants heard one of four numbers: 1, 2, 3 or 4 (Fig. 1D). These mapped to the four buttons of a button-box, and the participants pressed the corresponding button. Participants executed a total of 10 easy and 10 hard blocks. Numbers presented across trials of a block were random in sequence. Difficulty was manipulated using two parameters. The first was the response time window. In easy trials, participants had 1.5 s to press the corresponding button. In hard trials, this window was shortened to 0.5 s (failure to respond within the allotted time or pressing an incorrect button resulted in negative feedback with a brief sound; there was no positive feedback). The second parameter was intertrial interval. The intertrial interval was reduced from 1.5 to 0.5 s on hard trials. This forced participants to be in a state of greater alertness and preparation on hard blocks.

### MRI data acquisition and analysis

MRI data were acquired on a 3 T Siemens Magnetom scanner using a 12-channel phased array head coil. The imaging protocol used a sequential descending T_2_*-weighted gradient-echo planar imaging (EPI) sequence with the following parameters: repetition time of 2000 ms, echo time of 30 ms, 32 oblique slices with 3 mm thickness and a 0.75 mm interslice gap, in-plane resolution of 3.0 mm × 3.0 mm, a matrix of 64 × 64, field of view (FOV) of 192 mm and a flip angle of 78°. T_1_-weighted magnetization prepared rapid gradient echo structural images were acquired with 1.0 mm slice thickness, 1.0 mm × 1.0 mm × 1.0 mm isometric voxel resolution, FOV of 256 mm, and 176 slices. We tried to cover the whole brain other than the brainstem and inferior cerebellum.

Analysis was done on SPM12 using automatic analysis (aa) v.5.^66^ For preprocessing, functional images were initially slice time corrected using the middle slice as the reference, realigned, then normalized to the Montreal Neurological Institute (MNI) template using the Diffeomorphic Anatomical Registration using Exponentiated Lie algebra (DARTEL) toolbox and co-registered with the structural T_1_-weighted images. During normalization, EPI images were resampled to a 2 mm × 2 mm × 2 mm resolution and smoothened using an 8 mm full-width at half-maximum Gaussian kernel.

A general linear model was used. Task blocks were modelled as epochs whose lengths were the duration of the respective blocks. Easy and difficult blocks were modelled separately. The button-press response at the beginning of each block was modelled as an epoch whose duration was the interval between participants being prompted to press when they were ready for the next block and the time when they pressed the button. Movement parameters were added to the general linear modes as covariates of non-interest. Regressors and covariates were convolved with the standard haemodynamic response function and entered into the general linear model. During the general linear model, the data were high-pass filtered at a cut-off period of 128 s. Contrast estimates from each participant were entered into a group-level analysis. Unless specified otherwise, all whole-brain results have been corrected for multiple comparisons using a false-discovery rate threshold of *P* < 0.05.

#### Regions of interest

**Figure 2:**
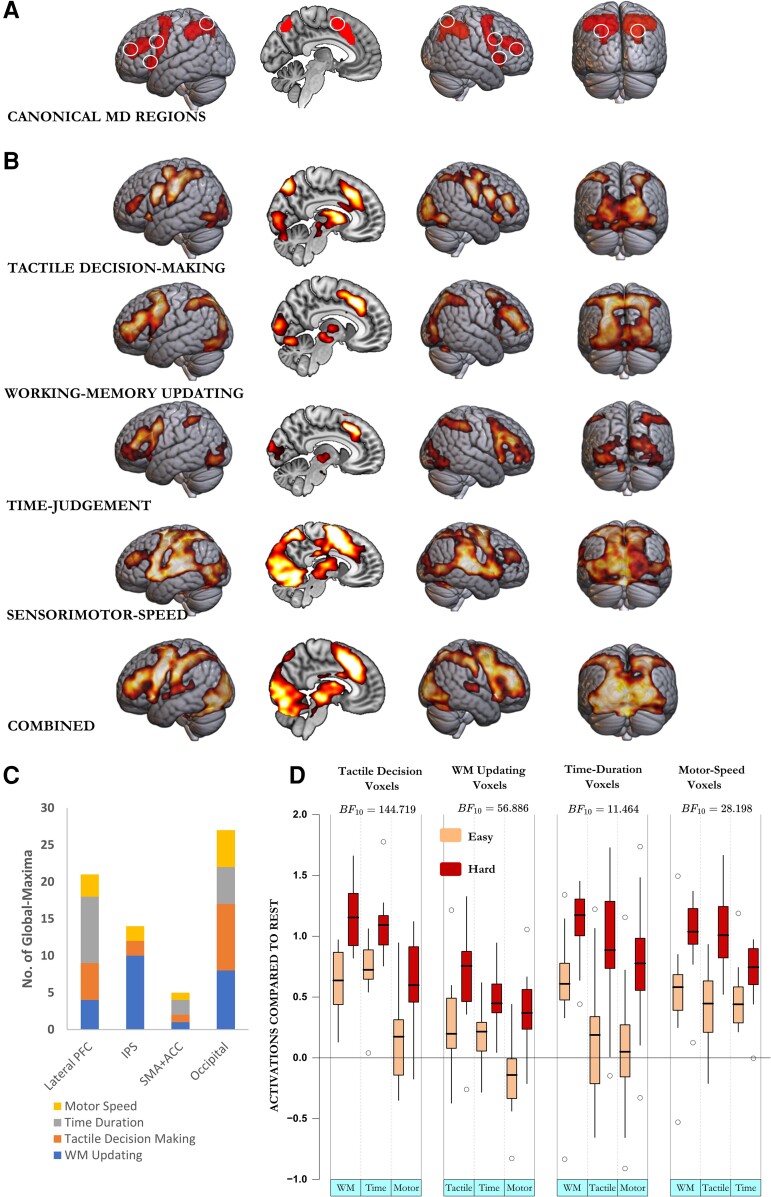
**Multiple demand regions, whole-brain activation, global maxima and occipital voxel response of the blind across the four diverse cognitive demands.** (**A**) Multiple demand (MD) regions include prefrontal regions extending from the inferior frontal junction along the inferior frontal sulcus and middle frontal gyrus up to the anterior prefrontal cortex, frontal operculum extending into the anterior insula, frontal eye fields, a blob extending from the pre-supplementary motor area to the anterior cingulate cortex, and parietal regions along the intraparietal sulcus. White circles show the location of spherical regions of interest used in later analyses (shown in Figs 3 and 6). (**B**) Combined across the four tasks, almost the entire occipital cortices in the blind, along with the frontoparietal MD regions, showed higher activation during hard compared with easy blocks. This was also the case in each of the four tasks individually. (**C**) Global maxima (or the voxel most intensely activating to control demands) occurred most frequently in these occipital regions. Lateral prefrontal cortex (PFC) in this analysis included the inferior frontal sulcus, inferior frontal junction, middle frontal gyrus, anterior prefrontal cortex, frontal operculum and anterior insula, bilaterally. IPS included the intraparietal sulcus, superior parietal lobule and the upper half of the inferior parietal lobule. The SMA and ACC included the pre-supplementary motor area, anterior cingulate cortex and any global maxima on the medial side of the prefrontal cortices. (**D**) Response of voxels that activated intensely to one control demand, to the remaining three control demands. BF_10_ represents the likelihood of these voxels activating across the three remaining control demands. WM = working memory.

Frontoparietal MD regions of interest (ROIs) were made as spheres of 10 mm diameter at respective coordinates that correspond to peaks of activation in response to multiple control demands (rough locations shown in Fig. 2A). The coordinates were taken from Dosenbach *et al*.^67^ and Duncan.^32^ These ROIs included the anterior prefrontal cortex (APC), pre-supplementary cortex (SMA), inferior frontal sulcus (IFS), intraparietal sulcus (IPS) and anterior insula (AI) bilaterally. We used two visual ROIs. These were extrastriate cortex (ESV) taken from Fedorenko *et al*.^37^ and the primary visual cortex (V1) made using the JuBrain Anatomy Toolbox.^68^ Frontoparietal MD ROIs were spherical ROIs, whereas visual ROIs were mask ROIs. Auditory cortex ROIs included Te1.0, Te1.1, Te1.2, Te3 and superior temporal sulcus (STS) 1 and 2. These, too, were taken from the JuBrain Toolbox. All ROI analyses (other than the one specified below) were done in the normalized space, and the same ROIs were used across all participant groups.

#### Delineating the occipital lobe voxels sensitive to each control demand

To test reliably for a key sign of MD regions (the same voxels activate in response to all control demands), we delineated the occipital lobe voxels most sensitive to each of the four control demands in every blind participant individually. We used JuBrain Anatomy Toolbox’s occipital cortex mask unnormalized into the native space of each blind subject. For each control demand, using unsmoothed images, we then located occipital cortex voxels that showed hard > easy significance at uncorrected *P* < 0.001. For some participants who did not have any voxels surviving this threshold, we used a lower threshold of *P* < 0.01. These voxels were then converted into functional ROIs using the MarsBaR toolbox (https://marsbar-toolbox.github.io). We thus had four functional ROIs for each participant corresponding to the set of occipital voxels activating in response to each of the four demands. We then examined how a set of voxels that activated maximally in response to one control demand (e.g. tactile decision task) responded to the remaining three control demands (WM-updating, time-duration judgment and sensorimotor speed).

We compared activations of ROIs across easy and hard blocks using Bayesian analyses. In all Bayesian analyses, we used priors that were default to JASP (v.0.17.3; https://jasp-stats.org/), which distributed priors uniformly across models. We use Bayes factor (BF) BF_10_ > 3 as evidence for the alternative hypothesis and BF_01_ > 3 as evidence for the null hypothesis.

### Connectivity analyses

#### Functional connectivity preprocessing

Functional connectivity analyses were done with the CONN toolbox^69^ (v.21.a) of SPM v.12. To mitigate movement-related variance, the smoothened functional data underwent realignment using the realign and unwarp procedure of SPM. This process involved co-registering all scans to a reference image (the first scan of the first session) via a least-squares approach using a six-parameter (rigid body) transformation^70^ and resampling using b-spline interpolation. Scans with framewise displacement >0.5 mm or global blood oxygenation level-dependent (BOLD) signal changes >3 SD were identified as potential outlier scans using ARtifact Detection Tools.^71-73^ A reference BOLD image was computed for each subject by averaging all scans, excluding outliers. Furthermore, functional data underwent denoising through ordinary least-squares linear regression to eliminate potential confounding effects related to motion and physiological artefacts. This denoising procedure followed the CompCor noise-reduction method^74^ using the standard pipeline in CONN.^71^ This included regressors such as white matter time series, CSF time series, 12 motion parameters along with their first-order derivatives, and the identification and removal of outlier scans. To prevent spurious inflation of functional connectivity estimates owing to task-induced coactivations,^75-77^ task-induced effects and their first-order derivatives (eight factors) were also modelled and regressed out from each functional run. Subsequently, a high-pass filter was applied, retaining frequencies of >0.01 Hz.^78^ The effective degrees of freedom of the BOLD signal after denoising were estimated to fall within the range of 1430.4–1872, with an average of 1589.1, across all subjects.^71^

#### First-level connectivity analysis

Seed-based connectivity maps were generated by characterizing functional connectivity spatial patterns across all experimental conditions, with a seed area using 164 brain networks defined by independent component analysis of the Human Connectome Project dataset^69^ (HCP-ICA) and Harvard–Oxford atlas ROIs^79^ as seed regions. The strength of functional connectivity was quantified through Fisher-transformed bivariate correlation coefficients derived from a weighted general linear model,^80^ computed separately for each seed area and target voxel, modelling the association between their BOLD signal time series. We used weighted seed-based connectivity maps generated by the CONN toolbox, wherein weights are boxcars representing task blocks convolved with the canonical haemodynamic response function.

#### Group-level connectivity analysis

Group-level analyses were conducted using a general linear model.^80^ In this approach, a separate general linear model was computed for each individual voxel, where the dependent variables were the first-level connectivity measures at that voxel, and the independent variables included groups or other subject-level identifiers. Multivariate parametric statistics with random effects across subjects and sample covariance estimation across multiple measurements were used to evaluate voxel-level hypotheses. Inferences were made at the level of individual clusters (groups of adjacent voxels), based on parametric statistics from Gaussian random field theory.^81,82^ The results were thresholded using a combination of a cluster-forming *P* < 0.001 voxel-level threshold and a false-discovery rate-corrected *P* < 0.05 cluster-size threshold.^83^

Group-level analyses were carried out using two seed regions. The prefrontal seed included the inferior frontal sulcus and the middle frontal gyrus extending up to anterior prefrontal regions specified as the frontoparietal (left and right lateral PFC) ROIs taken from the HCP-ICA networks.^69^ These correspond to the lateral prefrontal MD regions. The occipital seed included medial and lateral occipital regions [Brodamm area (BA) 17, 18 and 19] specified as the visual medial and visual occipital networks, selected from the HCP-ICA networks. These are the regions showing MD-like activation in the blind.

## Results

### Occipital cortices of the blind activate in response to multiple demands

Accuracies across all tasks and participant groups were >80%. Both reaction times and error rates were higher in difficult tasks (for details, see Supplementary Fig. 1). Figure 2A shows the pattern of frontoparietal MD activation typically seen in response to any control demand.^34,37^ These were expectedly active for each of the four control demands in the blind. However, and crucially, in these participants, occipital regions were additionally active for each of these four control demands and when combined across them (Fig. 2B). These regions included medial and lateral occipital regions (BA 17, 18 and 19) bilaterally, extending into inferior temporal regions (BA 37, TE1m and TE1p) laterally and into posterior parietal regions anteriorly. Activation of these regions was also discernible in most participants individually (Supplementary Fig. 2A and B). Not only did occipital regions of the blind activate in response to multiple demands, but they were, in a sense, the best MD regions, because across the various control demands, voxels that activated most intensely in response to the control demand, or the global maxima, were most frequently present in them (Fig. 2C).

### The same voxels within the occipital cortices of the blind activate in response to multiple demands

A key feature of MD regions is the activation of the same set of voxels in response to different control demands.^34,37^ To see whether visual regions of the blind showed this characteristic, for each subject we used the response to one control demand (e.g. WM-updating) to delineate the set of most intensely activating occipital voxels. This was done on their unnormalized and unsmoothed images. We then recorded the response of this functional ROI to the remaining three demands (i.e. tactile decision, time judgement and sensorimotor speed). We repeated this procedure for each of the four control demands. Functional ROIs created using one control demand always showed significant responses to the remaining three control demands (Fig. 2D). In each of the four kinds of ROIs, Bayesian repeated-measures ANOVA showed that the model that included type of control and difficulty level (easy or hard) as factors was significantly better at explaining the data than the null model (BF_10_ > 28). The Bayes factors for including difficulty level in the analyses with WM, tactile, time-duration and sensorimotor-speed ROIs were 56, 145, 11 and 28, respectively.

### Occipital cortices of the sighted do not activate in response to multiple demands

**Figure 3:**
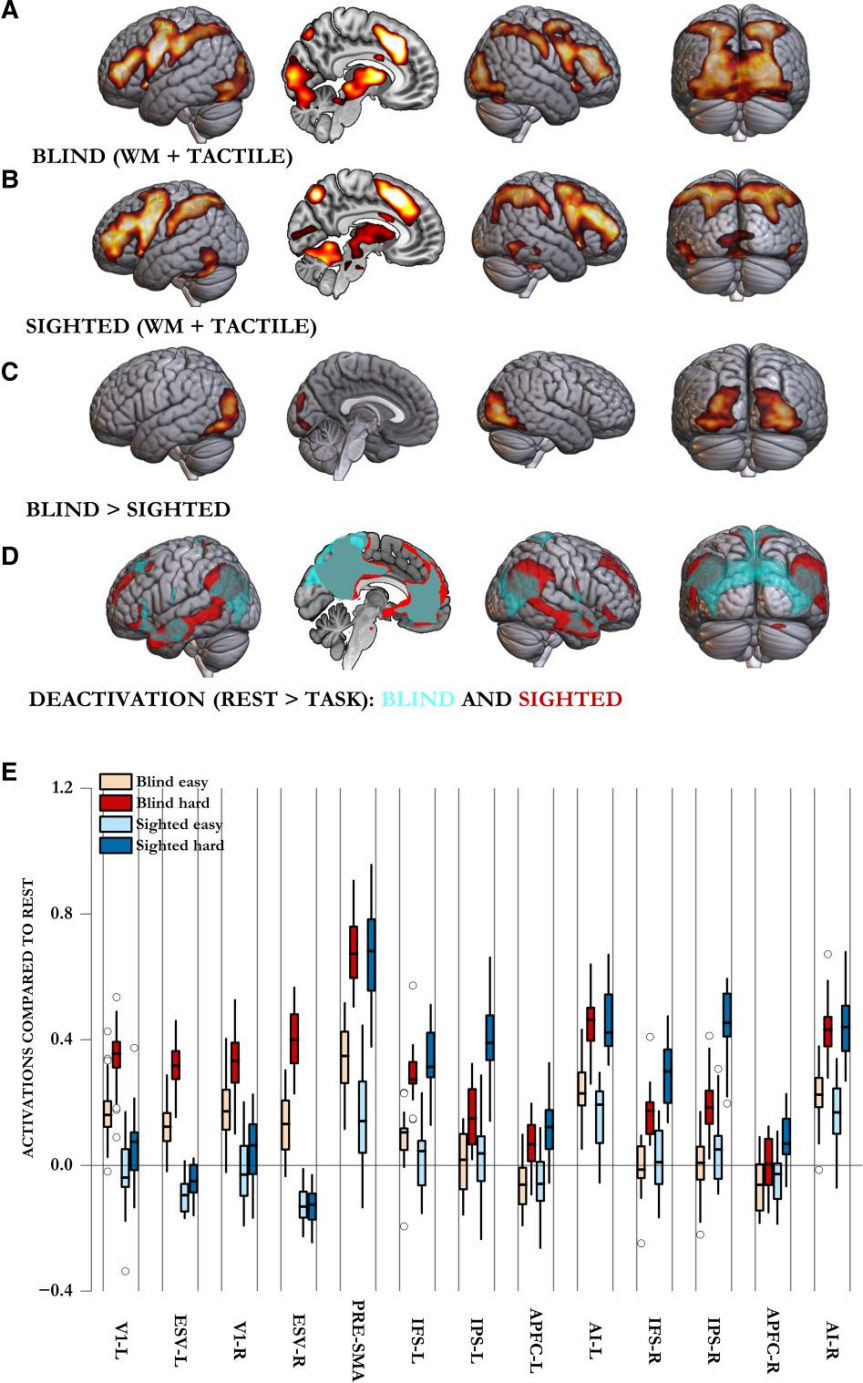
**Comparison of blind and sighted participants across tactile decision-making and auditory working memory-updating tasks.** Although both blind and sighted participants activated their canonical frontoparietal MD regions during the hard blocks of these tasks (**A** and **B**), only blind participants activated their occipital regions. Interestingly, sighted participants did activate two visual regions (BA 37 in posterior inferior temporal lobes and small clusters in V1) across the control demands of these non-visual tasks. These, however, were lower in intensity and spread than in blind participants. (**C**) Whole-brain contrast of blind versus sighted participants showed that the two control demands activated visual occipital regions of the blind significantly more than those of the sighted. (**D**) Sighted participants, unlike blind participants, showed task-related deactivation of parts of their extrastriate occipital regions. (**E**) Activations compared with rest across occipital and frontoparietal MD regions. Occipital regions in the blind activated significantly more during tasks than rest and during hard compared with easy blocks. These regions in the sighted either deactivated (ESV) or did not activate more than rest (V1 during easy blocks). Only during the hard blocks did V1 activate slightly more than the rest. In the sighted, hard blocks did not elicit significantly higher activity than easy blocks. Although both blind and sighted participants showed frontoparietal MD activation during hard compared with easy blocks, this activation was lower in the blind. AI = anterior insula; APFC = anterior prefrontal cortex; ESV = extrastriate visual cortex; IFS = inferior frontal sulcus; IPS = intraparietal sulcus; L = left; MD = multiple demand; pre-SMA = pre-supplementary motor area; R = right; V1 = primary visual cortex; WM = working memory.

Sighted participants, in comparison, mainly activated their canonical frontoparietal MD regions across the two tasks they executed: auditory WM-updating and tactile decision-making (Fig. 3B). Interestingly, they did activate a visual region corresponding to BA 37 in posterior-inferior temporal lobes (TE1m and TE1p^67^), in addition to a small voxel cluster in V1. The region BA 37 has previously been observed to show MD-like activation and is likely to be an MD region even in the sighted.^34^ Most of the occipital lobes showed no control-related activation in the sighted. Instead, parts of the lateral occipital regions deactivated in response to task demands. Deactivation during task execution is a response by a different set of domain-general regions called the default mode regions.^84^ This set of regions is frequently anti-correlated with MD regions.^40^ The sighted participants deactivated some of their lateral occipital regions during task blocks compared with rest, whereas no visual region in the blind showed such a response (Fig. 3D). A direct contrast between sighted and blind participants in these two tasks showed that the blind participants had significantly higher bilateral activation of occipital regions to control demands (Fig. 3C).

The differences in the responses of occipital regions of the blind and the sighted were also evident in ROI analyses (Fig. 3E). We used anatomical ROIs corresponding to V1 and ESV cortices, in addition to frontoparietal MD ROIs that correspond to loci of maximal activation to control demands.^47,67,85^ In the blind, both ESV and V1 activated in response to tasks compared with the resting baseline (BF_10_ > 42 077 on one-sample Bayesian *t*-test), whereas in the sighted, ESV deactivated (BF_10_ > 100) while V1 showed minimal to no activation (BF_10_ < 6; for details, see Supplementary Table 1). Furthermore, only the blind showed unambiguous activation of their visual regions in the hard compared with easy blocks (BF_10_ > 27). In the sighted, there was either evidence for the null hypothesis that easy blocks do not have lower activation than hard blocks (ESV, right: BF_01_ = 5), or the evidence for the alternate hypothesis was ambiguous (BF_10_ < 2; for details, see Supplementary Table 2). We tested the response of visual ROIs across the blind and sighted participant groups using Bayesian repeated-measures ANOVA with blindness as the between-subject factor. The best model, by far, to explain the data was the one that took into account the effects of region, difficulty, blindness and an interaction between difficulty and blindness (BF_10_ compared with the null model was 3 × 10^69^). The Bayes factor for including the interaction between blindness and difficulty was 5.9 × 10^7^, and the Bayes factor for including the effects of blindness was infinite. Thus, the difference between the blind and the sighted was significant both in terms of overall activation (i.e. the blind activated these regions more than the sighted) and in terms of the effect of difficulty (i.e. the blind showed higher activation to hard compared with easy blocks).

### Frontoparietal multiple demand activation in the blind is attenuated compared with the sighted

In contrast to visual regions, both blind and sighted participants activated their frontoparietal MD regions, especially during hard blocks. Nonetheless, there was a difference between them. Visual regions turning into MD regions will mean that blind participants have additional MD regions. This predicts that they do not have to use their canonical MD regions as much as sighted participants. This is evident in Fig. 3E wherein the difference between activations across the easy and hard blocks is smaller in the blind compared with the sighted. We analysed this formally through a Bayesian ANOVA, with MD regions, Difficulty and Blindness as factors. The best model to explain the data included the effects of Region, Blindness, Difficulty, Blindness × Difficulty, Blindness × Region, Difficulty × Region and Blindness × Region × Difficulty (BF_10_ = 10^228^). Crucially, the Bayes factor for including a difference in the effects of difficulty across the blind and the sighted (Blindness × Difficulty) was 619. Although these analyses were done on spherical ROIs that correspond to peak activations in the frontoparietal MD regions, the results remain largely identical if we use mask ROIs that correspond to all frontoparietal MD regions (Supplementary Figs 3 and 4).

### Occipital regions of the blind were more connected to the frontoparietal multiple demand regions

**Figure 4:**
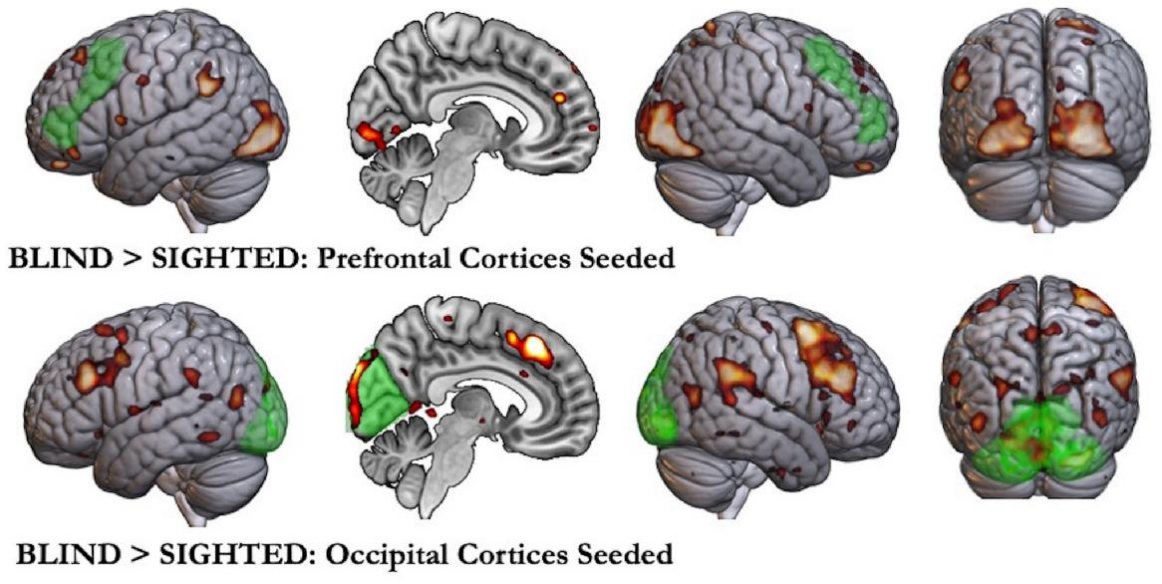
**Functional connectivity in the blind compared with the sighted.** Regions that showed higher functional connectivity in the blind compared with the sighted when prefrontal MD regions were seeded (*top*) and when occipital regions were seeded (*bottom*). Seeded regions are marked in green. The prefrontal seed region included the inferior frontal sulcus and the middle frontal gyrus extending up to anterior prefrontal regions. Occipital seed included medial and lateral occipital regions (Brodmann area 17, 18 and 19). Seeding prefrontal MD regions showed higher connectivity between them and visual occipital regions in the blind compared with sighted participants. Seeding occipital cortices showed higher connectivity in the blind compared with sighted participants, between them and key MD regions (middle frontal gyrus, inferior frontal sulcus, premotor regions, anterior insula, pre-supplementary motor areas and intraparietal sulcus) in addition to temporoparietal junctions. MD = multiple demand.

Not only did blind occipital regions activate like MD regions, but they were also more functionally connected to MD regions compared with sighted occipital regions. Seeding the occipital cortices showed significantly higher connectivity between them and the MD regions in the blind compared with the sighted (Fig. 4). Likewise, seeding MD-related prefrontal regions showed significantly higher connectivity between them and the occipital cortices in the blind compared with the sighted.

### Auditory regions of the blind do not activate reliably to the control demands of auditorily presented tasks

**Figure 5:**
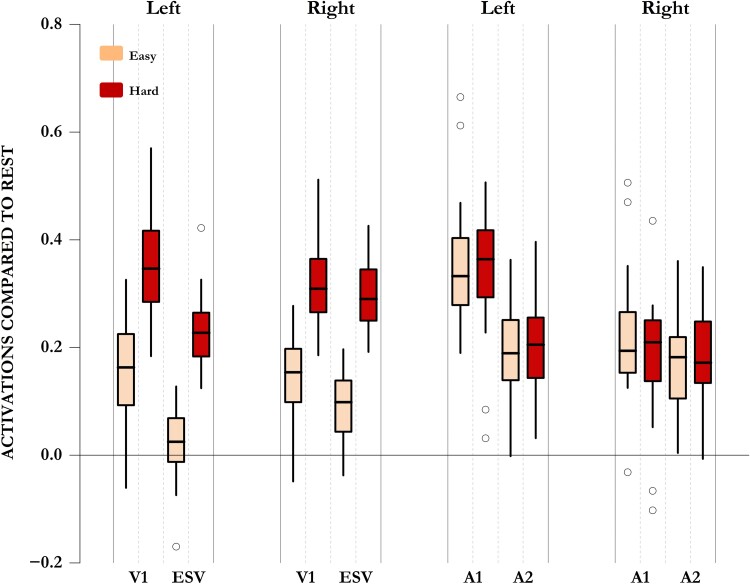
**Responses of visual and auditory regions of the blind to the auditorily presented tasks.** Responses of occipital and auditory regions of blind participants to easy and hard blocks of auditorily presented tasks (auditory WM-updating, time judgement and sensorimotor speed). Note that both V1 and extrastriate visual regions (ESV) activated intensely to control demands (evident in very high BF_10_ values from Bayesian paired *t*-tests in Table 1), whereas neither the primary nor the secondary auditory cortices showed any evidence of control-related activation. In these areas, there was significant evidence in favour of the null hypothesis that these regions did not activate to control demands (BF_01_ > 3; Table 1). A1 = primary auditory cortex; A2 = secondary auditory cortex; BF = Bayes factor; ESV = extrastriate visual cortex; V1 = primary visual cortex; WM = working memory.

For many accounts, occipital regions of the blind take up some other sensory role.^11,24,86^ Sensory regions during any task (e.g. visual regions in case of visual tasks) may activate during more demanding conditions owing to top-down inputs coming into them from higher control-related MD regions.^87^ But this is unlikely to explain the occipital activation in the blind across task demands involving diverse modalities (auditory, tactile and motor). An occipital region that is auditory cannot be expected also to activate in response to tactile control demands. Feedback as an explanation for the activation of occipital regions of the blind also becomes unlikely because, in contrast to these regions, the primary and secondary auditory regions of the blind did not activate in response to the control demands of auditorily presented tasks (Fig. 5). These auditory regions activated equally well during both easy and hard blocks compared with the resting baseline, showing their involvement in task-related sensory processing. However, these regions did not show increased activation during hard compared with easy blocks. Bayesian analysis showed significantly higher evidence in favour of the hypothesis that these auditory regions did not activate during hard compared with easy blocks (Fig. 5). Visual cortical activation in the blind in response to different control demands, therefore, cannot be a sensory processing response to increased control demands.

**Table 1:** Bayes factors of the activation of visual and auditory regions on hard blocks compared with easy blocks of auditorily presented tasks

ROIs	BF_10_^a^	BF_01_^b^
Left V1	468	0.002
Left ESV	2.1 × 10^6^	5 × 10^−6^
Left A1	0.2	5.6
Left A2	0.3	2.9
Right V1	1089	9 × 10^−4^
Right ESV	59 497	2 × 10^−5^
Right A1	0.1	7.3
Right A2	0.2	4.1

A1 = primary auditory cortex; A2 = secondary auditory cortex; BF = Bayes factor; ESV = extrastriate visual cortex; ROI = region of interest; V1 = primary visual cortex.

^a^BF_10_ shows the likelihood of the ROI showing higher activation on hard compared with easy blocks.

^b^BF_01_ shows the likelihood of the ROI not showing higher activation on hard compared with easy blocks.

### Auditory regions of the deaf do not activate to control demands

**Figure 6:**
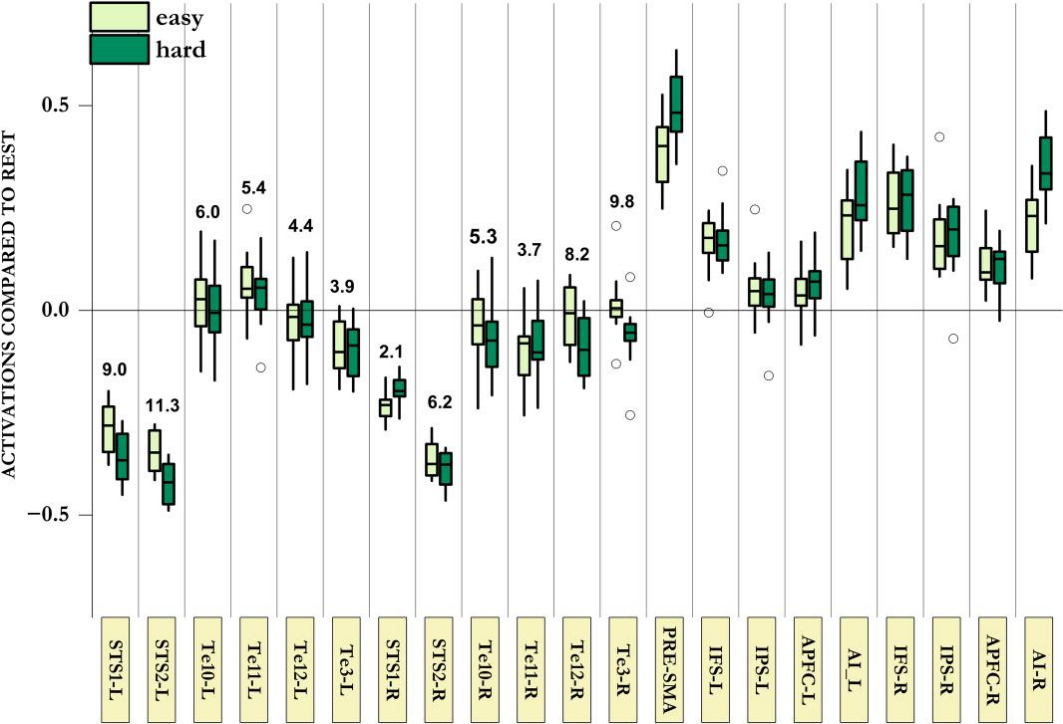
**Responses of primary and secondary auditory regions in the deaf during tactile decision-making and working memory-updating tasks.** Response of anatomically localized auditory regions and frontoparietal MD regions in the deaf across the two control demands (tactile decision-making and visual WM-updating). Note that none of the auditory regions showed any activation above the resting baseline during easy or hard task blocks, nor did any of these regions show higher activation during hard compared with easy blocks. Numbers above auditory ROI plots show the Bayes factor in favour of the null hypothesis (BF_01_) that hard and easy blocks did not differ in their activation levels. Most auditory regions showed significant evidence in favour of this null hypothesis (BF_01_ > 3). BF = Bayes factor; L = left; MD = multiple demand; R = right; ROI = region of interest; WM = working memory.

Unlike the visual cortices of the blind, deprived auditory cortices of deaf participants did not activate in response to the two control demands they were tested on (visual WM-updating and tactile decision-making). For all anatomical auditory ROIs, there was evidence in favour of the null hypothesis that these regions did not show higher activity during hard blocks (Fig. 6). Furthermore, as evident in Fig. 6, these auditory regions either did not activate or deactivated during task blocks compared with the resting baseline. This was very different from frontoparietal MD regions, most of which showed the expected activation above baseline (for details, see Supplementary Tables 3.1 and 3.2). Thus, auditory regions in the deaf are not MD regions by both criteria: they did not activate to control demands, and they did not activate above the resting baseline even in the hard task condition. Becoming MD regions, therefore, seems to be something unique to occipital regions of the blind and is not the fate of all deprived sensory regions.

## Discussion

We found that the entire occipital cortex in congenital and early blind participants behaved like MD regions and activated across highly diverse forms of control demands. Unlike sensory regions that may sometimes show increased activation to control demands during tasks in their modality (e.g. visual cortical activation during visual working memory^50^), occipital activation in the blind was not limited to control demands in one sensory modality. Instead, the same regions activated in response to auditory working memory, tactile decision-making, sensorimotor-speed and time-duration judgement demands. Furthermore, these occipital regions showed more intense activation to control demands during auditorily presented tasks than auditory regions themselves, precluding feedback to sensory processing regions as an explanation for their activation. The voxels maximally activating to control demands were most frequently present in occipital regions compared with any of the other MD regions. Blind participants possessing these additional occipital MD regions showed less control-related activation of their canonical frontoparietal MD regions than sighted participants who did not have these additional MD regions. Occipital regions of the blind were also more functionally connected with the canonical prefrontal MD regions. Unlike occipital regions of the blind, auditory regions of the deaf did not show any signs of MD regions, suggesting that occipital cortices might have a special predilection to become MD regions and that this fate might not be shared by other deprived sensory cortices.

Some authors have argued that visual cortical activation in response to tasks in other modalities is limited to higher visual regions that might be multisensory even in the sighted.^30,31,88^ Other accounts have noted that only parts of V1 show activation in response to higher cognitive demands^86^ (e.g. left V1 to language, right V1 to mathematics^29^). However, we found that all occipital regions, not only V1, activated bilaterally in response to multiple control demands.

Occipital cortices in the blind turning into MD regions parsimoniously explain most neuroimaging findings about them.^11,29,86^ MD regions are well known to activate in response to any task-relevant event, irrespective of sensory or motor modality.^35,36,54^ Experimental designs typically make the object of their study the most task-relevant event, and completing it is the goal of the task.^89^ In auditory tasks, for example, sound stimuli become the task-relevant events that are tied to some kind of goal completion. These can, therefore, be expected to activate frontoparietal MD regions in addition to occipital regions that have become MD regions. MD regions do not activate only in response to task-relevant events; patterns of activity across their voxels have information about any kind of task-relevant event, allowing decoding of any task-relevant information.^54^ Studies on visual occipital regions in the blind have started to show the same. Action goals, attended sensory information and task-relevant semantics have so far been shown to be decodable from these regions.^27,55,88^ Disrupting processing in the occipital pole using transcranial magnetic stimulation disrupted Braille reading more than disrupting processing in hand-related sensorimotor regions.^90^ This observation again cannot be explained in sensory terms but can easily be accounted for if occipital regions are involved in domain-general cognitive control.

Control-related activations in blind occipital regions have, in the past, been explained as resulting from feedback from control-related MD regions. Such accounts will initially need to posit the nature of goings-on in occipital regions that are purportedly being controlled by feedback inputs. Typically, the targets of feedback control inputs tend to be modality-specific sensory, motor or mnemonic representations/processes.^87,91-93^ For the same occipital voxel to activate from feedback during tasks involving different modalities, neurons related to these different modalities should be present in the same voxel. This is unlikely, because neurons engaged in a specific processing occur together in a region.^94-97^ Furthermore, control-related feedback should have activated auditory regions during auditory tasks, but no such activation was observed (Fig. 5), making it difficult to explain why such feedback activated occipital regions but not the auditory regions engaged in sensory processing.

The three well-characterized higher-cognitive networks are MD, default mode and language networks. Of these, only MD regions activate in response to diverse control demands. Language regions do not activate to control demands, and default mode network regions show task-related deactivations and deactivate to control demands.^84,98^ We found that almost all occipital regions in the blind (BA 17, 18 and 19) activated bilaterally in response to different control demands, exactly like frontoparietal MD regions. There were no foci of task-related deactivation in these regions (Fig. 3D). Although our results suggest that visual occipital regions of the blind become MD regions and not some default mode network-like regions, it remains unclear whether some of these also behave like language regions. In the frontal lobes, language and MD regions remain separate even if their voxels interdigitate.^98-100^ Although there is some evidence for the same in blind visual cortices,^101^ it remains unclear whether the same regions in blind occipital regions would activate in response to both cognitive control and language demands.

The fact that all core visual regions in the blind show MD response and that some parts of these continue to show varying levels of MD response in the sighted (Fig. 3B) raises the possibility that becoming MD regions might be the default developmental trajectory of these regions. Visual inputs either shunt or build upon this trajectory. This is also suggested by the pattern of resting-state functional connectivity of visual regions in sighted infants, which more closely resembles blind compared with sighted (but blindfolded) adults.^102^ The visual regions of both sighted infants and blind adults show increased connectivity with control-related prefrontal MD regions. In contrast, the visual regions of sighted adults show stronger connectivity with sensory and motor regions.

An interesting observation in this regard comes from a child with no frontal lobes.^103^ Seeding her remaining parietal MD regions showed higher resting-state connectivity between them and the visual regions, something not shown by sighted participants, suggesting that visual regions might become part of MD regions in sighted people with extensive damage to MD regions. Although our study showed a predilection of visual regions for developing into MD regions, there is at least one case report suggesting that prefrontal regions might take a visual role in exceptional circumstances. A patient with hydranencephaly, who had no cortical regions other than the prefrontal cortices, could not only make use of visual information but also showed signs of visual awareness (Subject 3).^104^

Occipital regions having a default developmental trajectory towards becoming MD regions also explains why sighted participants showed cognitive-control activation, albeit much limited, in certain visual–occipital regions during non-visual tasks and in visual regions during visual tasks.^37^ Sighted participants activated their posterior inferior temporal region (BA 37), exactly like blind participants, to control demands of auditory and tactile tasks. Previous studies have also noted activation of this region in response to the control demands of non-visual tasks.^34^ Interestingly, we also found clusters of voxels in V1 of the sighted participants that activated to control demands during non-visual tasks, suggesting that some islands might continue to activate to control demands even in the sighted.

Extrastriate visual cortical regions are well known to activate to different control demands during visual tasks.^35,37^ Given that these activations were seen during visual tasks, they were not interpreted as a core MD region but as a visual region activating in response to feedback control inputs during visual tasks. However, auditory regions do not activate consistently to control demands during auditory tasks (Fig. 5). It is, therefore, possible that even the activation of the visual cortex to control demands during visual tasks is something unique amongst sensory cortices. The activation of these same regions in the blind to different control demands suggests that these regions might have a predilection for MD-like response. In the blind, these show MD response to all tasks, whereas in the sighted, they show this response only during visual tasks.^37^

Becoming MD regions is not a general response of all deprived sensory regions. Auditory regions of the deaf did not activate to control demands. Perhaps these regions preferentially become language regions owing to the intimate connection between hearing and language. Non-overlap of language and multiple demand regions has been suggested.^98-100^ This issue, nonetheless, awaits further investigation.

## Supplementary Material

awae187_Supplementary_Data

## Data Availability

Data can be requested from the corresponding author. Data will be made publicly available after we finish our ongoing analyses.
