# Supplementary material for: The visual cortex in the blind but not the auditory cortex in the deaf becomes multiple-demand regions
Source: Brain. 2024 Jun 12;147(10):3624–37. doi: 10.1093/brain/awae187 (PMC11449128; doi:10.1093/brain/awae187)
Supplement: awae187_Supplementary_Data [file awae187_supplementary_data.pdf]

Supplementary Material

Behavior

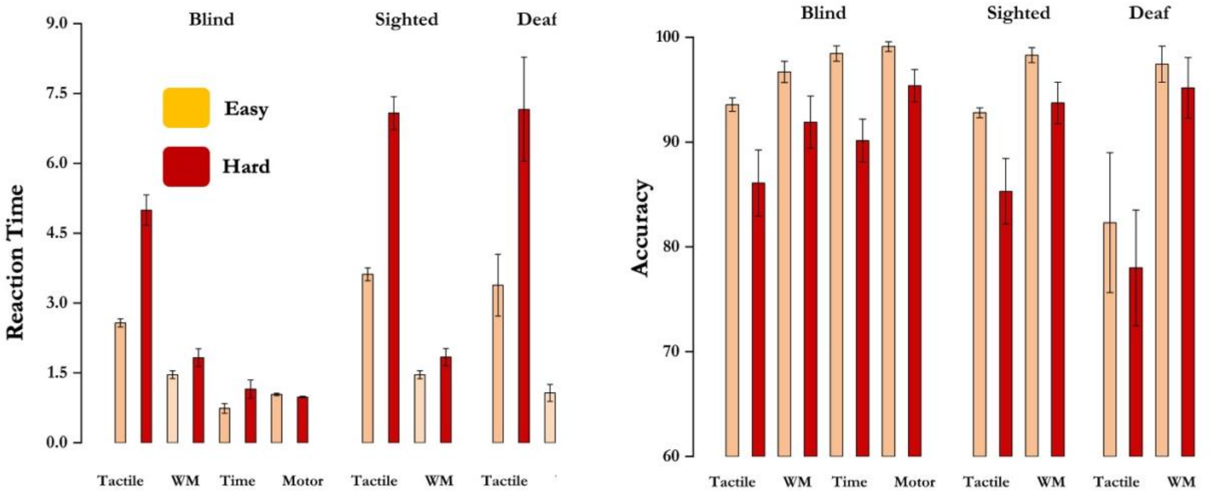

Fig.S1. Behavior of the blind, deaf and sighted control group. Accuracies were lower and RTs higher on the hard blocks of all tasks. Motor-speed task was different in that the harder blocks required faster responses, hence lower RTs on the hard blocks of this task. Error-bars represent 95% confidence intervals.

As evident in Figure 1, both blind and sighted participants were slower and less accurate on hard compared to easy blocks across in all tasks. We compared behavior of the blind and the sighted across the two tasks that sighted participants executed and the two difficulty-levels of these tasks [2 x 2 x 2 repeated measures ANOVA with task type, tactile decision-making and WM updating, and task difficulty (easy, hard) as within-subject factor and sightedness (blind, sighted) as between-subject factor on the mean accuracies and RTs]. There was an effect of task difficulty on accuracy, $F(1,41) = 93$, $p < 0.001$. Accuracy did not differ between blind and sighted participants across these tasks (mean difference of -0.005, SE = 0.007, $t(1,41) = -0.739$, $p = 0.43$ (Holm-adjusted)). There was an effect of task difficulty on RTs, $F(1,41) = 890$, $p < 0.001$. Blind participants were faster than sighted on tactile decision-making task [mean difference of -1.594, SE = 0.119, $t(1,41) = -13$, $p <0.001$ (Holm-adjusted)], and slowed down less on harder blocks [mean difference of -2.082 SE= 0.141, $t(1,41) = -15$, $p < 0.001$ (Holm-adjusted)]. RTs did not differ between these groups on WM updating task [mean difference of -0.006, SE = 0.109, $t(1,41) = -0.05$, $p = 0.96$ (Holm-adjusted)].

We analyzed behavior of deaf participants across the two tasks they executed and two difficulty levels of these tasks [2 x 2 repeated measures ANOVA with task type, tactile decision-making and WM updating, and task difficulty (easy, hard) on their performance of accuracy and RTs]. There was an effect of task difficulty on accuracy, $F(1,9) = 6.3$, $p=0.033$ as well as an effect of difficulty on RTs, $F(1,9) = 179$. Deaf participants were less accurate on the harder blocks of tactile decision-making task [mean difference of -0.043, SE = 0.017, $t(1,9) = -2.532$, $p = 0.042$ (Holm-adjusted)],

but not on WM updating task [mean difference of -0.023, SE = 0.017, t (1,9) = - 1.329, p = 0.201)]. Again, deaf participants were slowed down in the harder blocks of tactile decision-making task [mean difference of -3.775, SE = 0.192, t = -19,695 p < 0.001(Holm-adjusted)], but not on WM updating task [mean difference of -0.148, SE = 0.192, t (1,9) = -0.773, p = 0.450 (Holm-adjusted)].

Individual Blind Participant Images

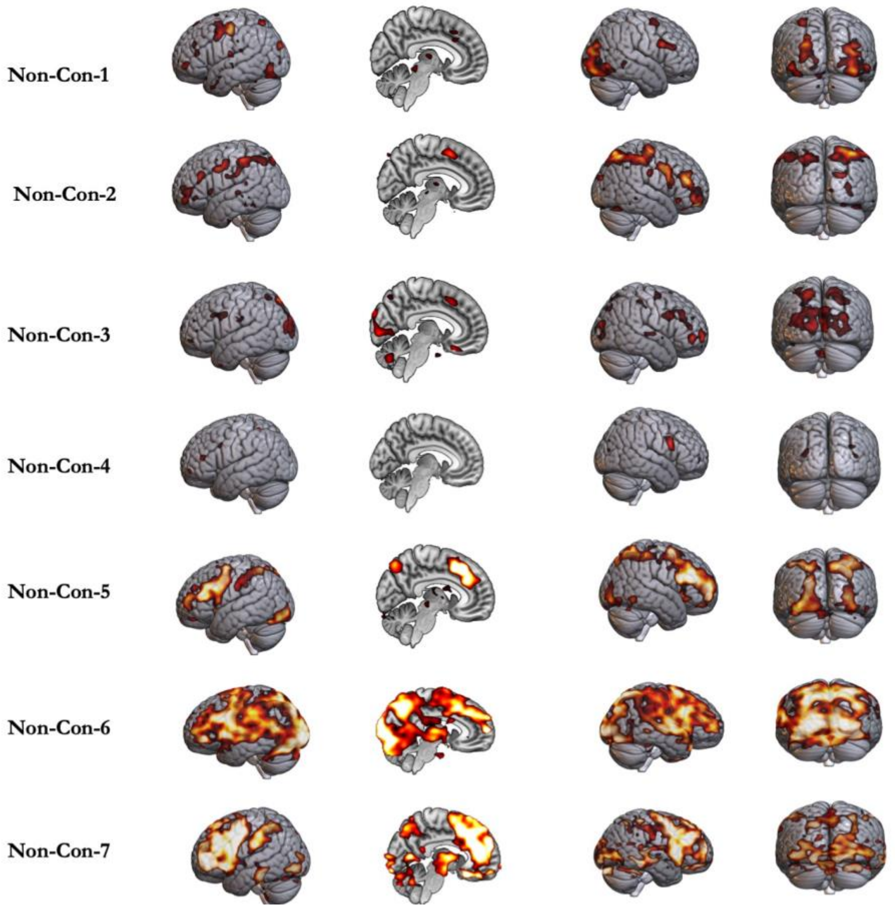

Fig. S2a. Whole brain results of individual non-congenitally blind participants.

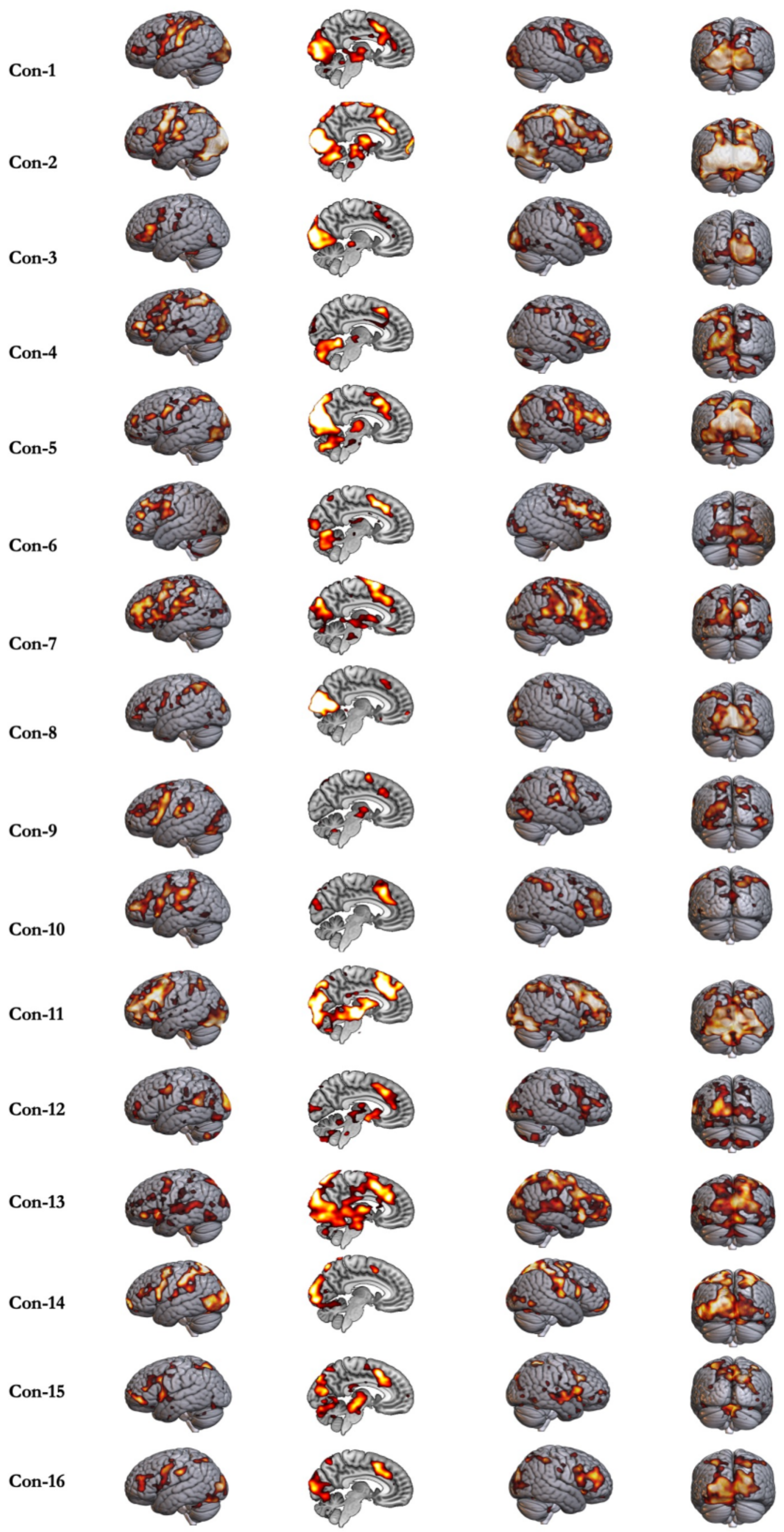

Fig. S2b. Whole brain results of individual congenitally blind participants.

Sighted vs Blind

Table S1. Bayes Factors for visual regions activating and deactivating in easy and hard conditions compared to the resting baseline.

			Blind		Sighted	
			Activation[1] BF_{10}	Deactivation[2] BF_{10}	Activation[1] BF_{10}	Deactivation[2] BF_{10}
**V1**	**Left**	**Easy**	230396	0.015	0.1	0.7
		**Hard**	1.5×10^{10}	0.007	5.9	0.1
	**Right**	**Easy**	55185	0.017	0.1	0.5
		**Hard**	2.7×10^{9}	0.008	3.5	0.1
**ESV**	**Left**	**Easy**	515039	0.014	0.01	144681
		**Hard**	1.2×10^{13}	0.005	0.06	100
	**Right**	**Easy**	42077	0.018	0.01	4.9×10^{6}
		**Hard**	5.4×10^{12}	0.005	0.01	7.9×10^{6}

1 Likelihood of the region activating during task conditions (easy, hard) compared to the resting baseline.

2 Likelihood of the regions deactivating during task conditions compared to the resting baseline

Table S2. Bayes Factors for visual regions showing higher activation (BF_{10}) or not showing higher activation (BF_{01}) in hard compared to easy conditions

		Blind		Sighted	
		BF_{10}[a]	BF_{01}[b]	BF_{10}[a]	BF_{01}[b]
**V1**	**Left**	51	0.015	1.8	0.5
	**Right**	28	0.04	2	0.9
**ESV**	**Left**	27634	3.6×10^{-5}	1.1	0.5
	**Right**	43273	2.3×10^{-5}	0.2	5

a Likelihood of the region showing hard>easy activation on tactile and WM updating tasks.

b Likelihood of the region not showing hard>easy activation on tactile and WM updating tasks

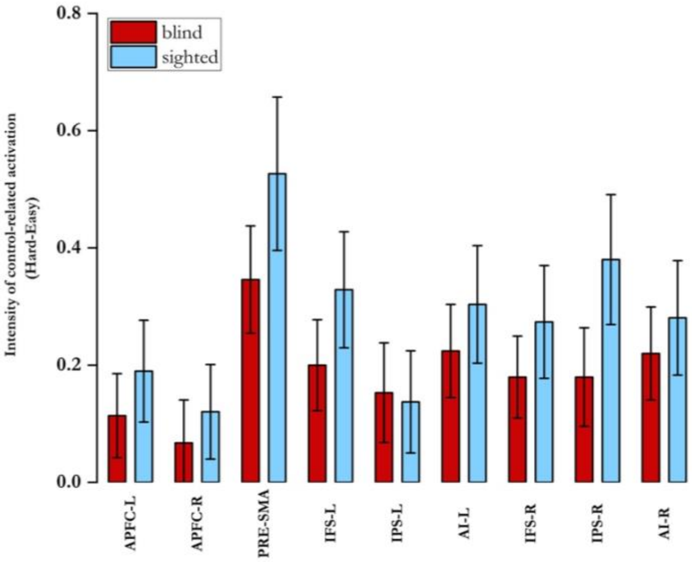

Fig. S3. Frontoparietal MD activation in blind and sighted. Beta values from easy conditions are subtracted from those in hard conditions for both tactile and WM updating tasks.

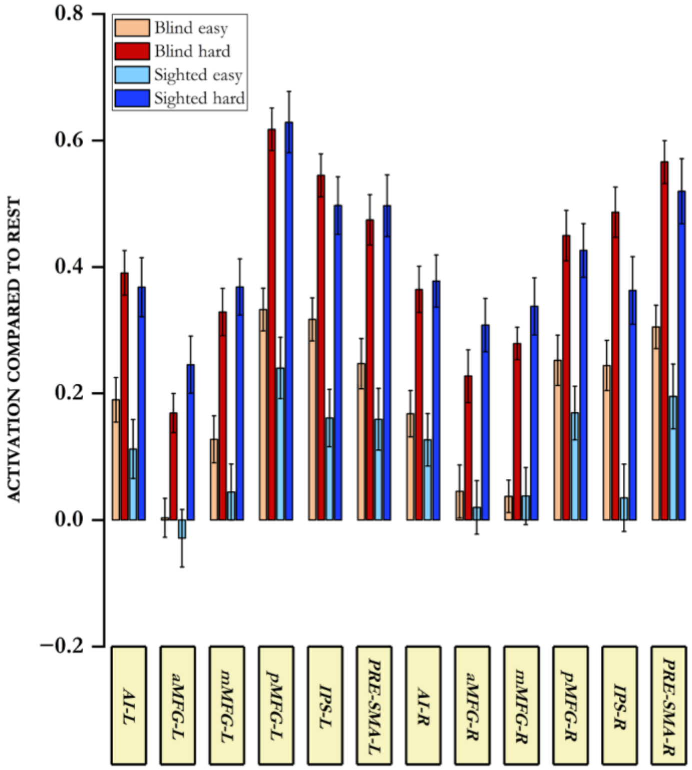

Fig S4. Activation of frontoparietal MD mask rois across the easy and hard conditions in blind and sighted participants. Note that the results are largely identical to that of frontoparietal spherical ROIs shown in Figure 3e of the main paper.

Deaf fMRI

Deaf participants did not show widespread activation of MD regions in the tactile decision-making task or the WM-updating tasks even at a lenient threshold of uncorrected p = 0.01 (Figure S5). We therefore compared the hard blocks to rest. This showed activations in MD regions along with extrastriate visual cortices. However, even here there was no auditory cortex activation. In fact, auditory regions predominantly deactivated during both easy and hard conditions compared to rest (Table 3.1). In contrast, MD regions predominantly activated compared to rest (Table 3.2).

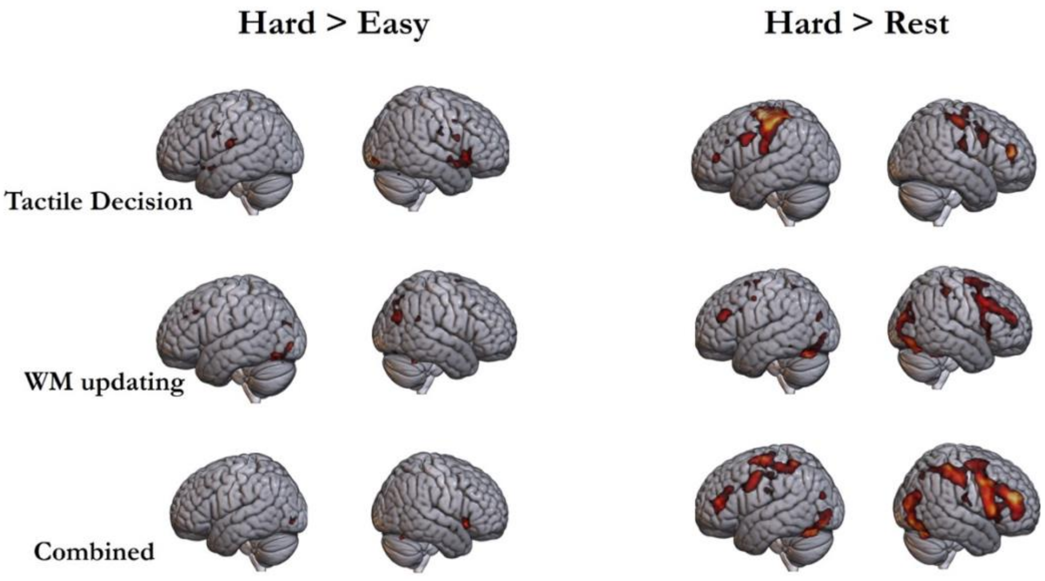

Fig. S5. Whole brain activation in the deaf group across tactile and WM updating demands. Deaf participants did not reliably activate MD regions on hard compared to easy blocks. They however did activate MD regions on hard blocks compared to rest periods. However, even here they did not activate auditory regions. Images are thresholded to uncorrected $p < 0.01$.

Table S3.1 Bayesian Analysis of auditory ROIs activation/deactivation compared to resting baseline in the deaf group.

ROIs	BF_{act} [a]	BF_{deact} [b]
Left STS1 easy	0.01	159262.5
Left STS1 hard	0.01	811778.9
Left STS2 easy	0.01	3.7×10^6
Left STS2 hard	0.01	1.8×10^7
Left Te10 easy	0.6	0.2
Left Te10 hard	0.3	0.3
Left Te11 easy	3.2	0.1
Left Te11 hard	1.4	0.1
Left Te12 easy	0.2	0.7
Left Te12 hard	0.2	0.7
Left Te3 easy	0.1	65.2
Left Te3 hard	0.1	43.7
Right STS1 easy	0.01	1.7×10^6
Right STS1 hard	0.01	476784
Right STS2 easy	0.01	1.2×10^7
Right STS2 hard	0.01	2.2×10^7
Right Te10 easy	0.2	1.1
Right Te10 hard	0.1	3.3
Right Te11 easy	0.1	17.2
Right Te11 hard	0.1	9
Right Te12 easy	0.2	0.6
Right Te12 hard	0.1	20.5
Right Te3 easy	0.5	0.2
Right Te3 hard	0.1	4

[a] Likelihood of ROIs activating to easy and hard conditions (easy, hard) across tactile and WM updating tasks compared to resting baseline.

[b] Likelihood of ROIs deactivating to easy and hard conditions (easy, hard) across tactile and WM updating tasks compared to resting baseline.

Table S3.2 Bayesian Analysis of fronto-parietal ROIs activation/deactivation compared to resting baseline in the deaf group.

**ROIs**	$\mathbf{BF_{act}}$ [a]	$\mathbf{BF_{deact}}$ [b]
Pre-SMA easy	135965.1	0.01
Pre-SMA hard	777708.7	0.01
Left IFS easy	599.9	0.03
Left IFS hard	1661.7	0.02
Left IPS easy	2.5	0.1
Left IPS hard	1	0.2
Left APFC easy	2	0.1
Left APFC hard	6.2	0.1
Left AI easy	1024.8	0.03
Left A1 hard	9278.1	0.02
Right IFS easy	8819.7	0.02
Right IFS hard	13205.2	0.02
Right IPS easy	203.4	0.03
Right IPS hard	145.5	0.04
Right APFC easy	223.9	0.03
Right APFC hard	186.7	0.03
Right AI easy	1930.8	0.02
Right AI hard	59106.6	0.02

[a] Likelihood of ROIs activating to easy and hard conditions (easy, hard) across tactile and WM updating tasks compared to resting baseline.

[b] Likelihood of ROIs deactivating to easy and hard conditions (easy, hard) across tactile and WM updating tasks compared to resting baseline.
